# Supplementary material for: Twin pair analysis uncovers links between DNA methylation, mitochondrial DNA quantity and obesity
Source: Nat Commun. 2025 May 12;16:4374. doi: 10.1038/s41467-025-59576-7 (PMC12069627; doi:10.1038/s41467-025-59576-7)
Supplement: Supplementary file 1 — Supplementary Information [file 41467_2025_59576_MOESM1_ESM.pdf]

Supplementary Information

Twin pair analysis uncovers links between DNA methylation, mitochondrial DNA quantity and obesity

Aino Heikkinen[1,2*], Vivienne F C Esser[3], Seung Hyuk T Lee[4], Sara Lundgren[1], Antti Hakkarainen[5], Jesper Lundbom[5,6], Juho Kuula[5,7], Per-Henrik Groop[8,9,10], Sini Heinonen[11,12], Sergio Villicaña[13], Jordana T Bell[13], Alice Maguolo[14], Emma Nilsson[14], Charlotte Ling[14], Allan Vaag[15,16,17], Päivi Pajukanta[4,18,19], Jaakko Kaprio[1], Kirsi H Pietiläinen[11,20], Shuai Li[3,21] & Miina Ollikainen[1,2*]

[1] Institute for Molecular Medicine Finland (FIMM), HiLIFE, University of Helsinki, Helsinki, Finland
[2] Minerva Foundation Institute for Medical Research, Helsinki, Finland
[3] Centre for Epidemiology and Biostatistics, Melbourne School of Population and Global Health, University of Melbourne, Melbourne, VIC, Australia
[4] Department of Human Genetics, David Geffen School of Medicine at UCLA, Los Angeles, CA, USA
[5] HUS Medical Imaging Center, Radiology, University of Helsinki and Helsinki University Hospital, Helsinki, Finland
[6] Institute for Clinical Diabetology, German Diabetes Center, Leibniz Center for Diabetes Research, Heinrich Heine University, Düsseldorf, Germany
[7] Public Health Promotion Unit, National Institute for Health and Welfare, Helsinki, Finland.
[8] Folkhälsan Institute of Genetics, Folkhälsan Research Center, Helsinki, Finland
[9] Research Program for Clinical and Molecular Metabolism, Faculty of Medicine, University of Helsinki, Helsinki, Finland
[10] Abdominal Center, Nephrology, University of Helsinki and Helsinki University Hospital, Helsinki, Finland
[11] Obesity Research Unit, Research Program for Clinical and Molecular Metabolism, Faculty of Medicine, University of Helsinki, Helsinki, Finland
[12] Department of Internal Medicine, Helsinki University Hospital, Helsinki, Finland
[13] Department of Twin Research and Genetic Epidemiology, King's College London, London, UK
[14] Epigenetics and Diabetes Unit, Department of Clinical Sciences in Malmö, Lund University Diabetes Centre, Scania University Hospital, Malmö, Sweden
[15] Department of Clinical Sciences in Malmö, Lund University Diabetes Centre, Scania University Hospital, Malmö, Sweden
[16] Copenhagen University Hospital, Steno Diabetes Center Copenhagen, Herlev, Denmark
[17] Department of Endocrinology, Skåne University Hospital, Malmö, Sweden
[18] Bioinformatics Interdepartmental Program, UCLA, Los Angeles, CA, USA
[19] Institute for Precision Health, David Geffen School of Medicine at UCLA, Los Angeles, CA, USA
[20] HealthyWeightHub, Endocrinology, Abdominal Center, Helsinki University Central Hospital and University of Helsinki, Helsinki, Finland
[21] Precision Medicine, School of Clinical Sciences at Monash Health, Monash University, Clayton, Victoria, Australia
* Corresponding authors

Supplementary Table 1. Number of monozygotic and dizygotic twin samples of mitochondrial DNA quantity, DNA methylation, or both, in each tissue.

	MZ pairs (individuals)	DZ pairs (individuals)	MZ individuals*	DZ individuals*
Adipose	71 (142)	4 (8)	6	1
Muscle	74 (148)	4 (8)	1	-
Total**	81 (162)	10 (2)	1	-

* Twins with missing co-twin data due to failing quality control during DNA methylation or mitochondrial DNA quantity data preprocessing.
** Total number of unique twin pairs or individuals with either adipose or muscle tissue sample or both available.

DZ = dizygotic; MZ = monozygotic

Supplementary Table 2. Epigenetic age estimates of the study cohort.

Epigenetic clock	Tissue	Mean (SD) , years
Horvath clock	Adipose	-0.2 (-8.7–7.7)
	Muscle	-0.3 (-7.6–6.2)
Hannum clock	Adipose	-0.2 (-8.0–6.5)
	Muscle	-0.1 (-5.3–5.0)
GrimAge	Adipose	-0.2 (-5.8–4.1)
	Muscle	-0.1 (-3.7–4.0)
PhenoAge	Adipose	-0.2 (-11.5–11.8)
	Muscle	-0.2 (-8.1–4.6)
DunedinPACE*	Adipose	1.7 (1.5–2.0)
	Muscle	1.4 (1.2–1.5)
MEAT	Muscle	0.2 (-8.5–7.3)

* unit: years/calendar year

Supplementary Table 3. Characteristics of the monozygotic twin pairs used in ICE FALCON analysis of SH3BP4 methylation, mtDNA quantity and obesity-related traits in adipose tissue.

Variable	Heavier co-twin (mean)	Leaner co-twin (mean)	Difference (Heavier-Leaner)	P-value**	N (MZ twin pairs)
cg19998400* (M-value)	0.82	0.79	0.03	8.0E−05	68
mtDNAq*	−0.06	0.02	−0.08	7.7E−05	71
Weight (kg)	91.60	77.84	13.76	5.4E−18	71
BMI (kg/m2)	31.93	27.32	4.61	4.6E−19	71
Waist circumference (cm)	104.38	91.96	12.42	3.6E−17	64
WHR	0.93	0.90	0.04	8.9E−05	33
Fat (%)	40.65	35.33	5.32	1.4E−13	71
Fat (kg)	38.36	28.79	9.57	3.0E−14	71
Fat-free mass (kg)	51.14	47.60	3.55	1.4E−07	71
Intra-abdominal fat (cm3)	1300.57	625.16	675.42	7.7E−04	21
Subcutaneous fat (cm3)	5933.65	3739.47	2194.18	3.8E−05	21
Adipocyte volume (mm3)	615	457	159	7.3E−08	60
Liver fat (%)	4.15	1.60	2.55	9.9E−04	42
Total cholesterol (mmol/l)	4.82	4.73	0.09	0.47	71
HDL (mmol/l)	1.47	1.64	−0.18	1.7E−04	71
LDL (mmol/l)	3.12	2.91	0.22	0.07	71
Triglycerides (mmol/l)	1.12	0.97	0.16	0.01	71
hsCRP (mg/l)	3.33	2.31	01.02	0.02	64
Adipsin (ug/l)	1.21	1.14	0.07	0.12	21
Adiponectin (ng/l)	2903.00	3694.00	-791.00	7.7E−04	21
ALAT (U/l)	29.20	24.93	4.27	0.03	71
ASAT (U/l)	28.94	27.27	1.68	0.40	71
BP (systolic) (mmHg)	134	134	0	0.74	69
BP (diastolic) (mmHg)	81	79	2	0.03	69
Fasting glucose (mmol/l)	5.82	5.55	0.27	0.11	69
Fasting insulin (mU/l)	9.50	7.28	2.22	3.0E−03	67
HOMA-IR	2.52	1.81	0.71	2.5E−03	64
Matsuda index	5.58	7.76	−2.18	7.8E−05	59
Leisure-time physical activity	2.91	2.92	−0.02	0.86	65
Sports activity	2.59	2.77	−0.18	0.12	66
Work activity	2.45	2.44	0.02	0.91	67
Total activity	7.98	8.17	−0.19	0.29	63
EAAHorvath*	0.14	−0.47	0.61	0.02	68
EAAHannum*	0.35	−0.49	0.84	5.5E−04	68
EAAGrim*	−0.43	−0.03	−0.39	0.05	68
EAAPheno*	0.26	−0.34	0.60	0.08	68
DunedinPACE*	1.70	1.69	0.00	0.80	68

* Estimates derived from adipose tissue

** Paired t-test (two-tailed)

ALAT = Alanine aminotransferase; ASAT = Aspartate aminotransferase; BMI = body mass index; DZ = dizygotic; EAA = Epigenetic Age Acceleration; HOMA-IR = Homeostatic model assessment for insulin resistance; hsCRP = high-sensitivity C-reactive protein; mtDNAq = mitochondrial DNA quantity; MZ = monozygotic; WHR = waist-to-hip ratio

Supplementary Table 4. Results from ICE FALCON analysis between mtDNA quantity and the identified CpG sites (n = 68 monozygotic twin pairs and 4 dizygotic twin pairs) in adipose tissue.

Formula	Coef*	Model 1			Model 2			Model 3			Change in coefficients	
		Est	SE	P	Est	SE	P	Est	SE	P	Est	P
mtDNAq (Y) cg19998400 (X)	βself	-0.363	0.059	**8.0E-10**				-0.384	0.058	**3.3E-11**	-0.021	0.492
	βcotwin				0.025	0.066	0.710	-0.097	0.058	0.098	-0.121	0.221
cg19998400 (Y) mtDNAq (X)	βself	-0.510	0.089	**8.8E-09**				-0.493	0.097	**3.7E-07**	0.014	0.634
	Bcotwin				-0.245	0.074	**0.001**	-0.048	0.070	0.491	-0.054	0.458
mtDNAq (Y) cg17468563 (X)	βself	-0.464	0.090	**2.3E-07**				-0.450	0.090	**5.6E-07**	0.017	0.592
	βcotwin				0.186	0.078	**0.018**	0.132	0.072	0.067	0.197	0.095
cg17468563 (Y) mtDNAq (X)	βself	-0.356	0.075	**2.2E-06**				-0.350	0.074	**2.3E-06**	0.006	0.763
	βcotwin				0.103	0.079	0.191	0.057	0.063	0.368	-0.046	0.585

*Standardized regression coefficient; β_{self} represents the association between twin's own X and Y variables whereas β_{cotwin} is the cross-twin cross-trait association i.e. the association between twin's own X variable with their co-twin's Y variable. X and Y in brackets indicate whether the variable was used as a predictor or an outcome, respectively. The p-values for models 1-3 were calculated from regression coefficients and standard errors using two-sided z-statistics. The change in coefficients was analysed using non-parametric bootstrapping. No multiple comparison adjustment was applied. Regression models were adjusted for age, sex, smoking, BMI and methylation beadchip and row. P-values<0.05 are bolded.

mtDNAq = mitochondrial DNA quantity

Supplementary Table 5. Results from the ICE FALCON analysis between the expression and methylation of *SH3BP4* and *DHRS3* (n=40 monozygotic twin pairs).

Formula	Coef*	Model 1			Model 2			Model 3			Change in coefficients	
		Est	SE	P	Est	SE	P	Est	SE	p.val	Est	P
SH3BP4 ~ cg19998400	βself	0.261	0.047	2.0E-08				0.267	0.047	1.3E-08	0.006	0.871
	βcotwin				-0.112	0.064	0.078	0.014	0.066	0.833	0.126	0.134
cg19998400 ~ SH3BP4	βself	0.652	0.186	4.7E-04				0.717	0.163	1.0E-05	0.065	0.494
	Bcotwin				0.031	0.162	0.846	-0.225	0.173	0.193	-0.256	0.299
DHRS3 ~ cg17468563	βself	-0.643	0.156	3.7E-05				-0.657	0.146	6.7E-06	-0.014	0.822
	βcotwin				-0.050	0.191	0.793	0.075	0.165	0.647	0.125	0.532
cg17468563 ~ DHRS3	βself	-0.296	0.072	3.7E-05				-0.306	0.071	1.5E-05	-0.010	0.696
	βcotwin				-0.008	0.078	0.916	-0.059	0.055	0.280	-0.051	0.581

*Standardized regression coefficient; β_{self} represents the association between twin's own X and Y variables whereas β_{cotwin} is the cross-twin cross-trait association i.e. the association between twin's own X variable with their co-twin's Y variable. The p-values for models 1-3 were calculated from regression coefficients and standard errors using two-sided z-statistics. The change in coefficients was analysed using non-parametric bootstrapping. No multiple comparison adjustment was applied. Regression models were adjusted for age, sex and smoking.

Supplementary Table 6. Associations between adipose tissue *SH3BP4* methylation and obesity-related traits.

Outcome	Coefficient	SE	P-value	FDR	N
Weight	0.246	0.070	4.1E-04	**0.002**	136
BMI	0.283	0.078	2.9E-04	**0.001**	136
Waist	0.300	0.065	4.0E-06	**3.5E-05**	122
WHR	0.123	0.062	0.050	0.079	64
Fat %	0.290	0.062	2.7E-06	**4.7E-05**	136
Fat (kg)	0.318	0.077	3.4E-05	**2.3E-04**	136
Fat-free mass	0.051	0.039	0.190	0.247	136
Ia. Fat[a]	0.458	0.099	3.6E-06	**4.1E-05**	42
Subcut. Fat[a]	0.352	0.086	4.5E-05	**2.6E-04**	42
Adipocyte volume	0.202	0.079	0.010	**0.022**	114
Liver fat %[a]	0.391	0.080	1.1E-06	**3.8E-05**	82
Total cholesterol	0.027	0.088	0.761	0.807	136
HDL	-0.189	0.068	0.006	**0.015**	136
LDL	0.059	0.092	0.523	0.631	136
Triglycerides[a]	0.222	0.090	0.013	**0.028**	136
hsCRP[a]	0.320	0.081	7.2E-05	**3.6E-04**	122
Adipsin	0.058	0.146	0.694	0.759	42
Adiponectin	-0.275	0.089	0.002	**0.006**	42
ASAT[a]	0.033	0.077	0.672	0.784	136
ALAT[a]	0.095	0.088	0.282	0.353	136
BP (systolic)	0.089	0.057	0.122	0.165	132
BP (diastolic)	0.206	0.063	0.001	**0.003**	132
Fasting glucose[a]	0.165	0.074	0.025	**0.046**	132
Fasting insulin[a]	0.233	0.097	0.016	**0.032**	128
HOMA-IR[a]	0.250	0.095	0.009	**0.021**	122
Matsuda[a]	-0.303	0.089	0.001	**0.002**	112
LTPA	-0.246	0.092	0.008	**0.019**	124
Sports activity	-0.178	0.088	0.042	0.074	126
Work activity	0.026	0.097	0.793	0.816	128
Total activity	-0.169	0.086	0.049	0.082	120
EAAHorvath	0.014	0.070	0.837	0.837	136
EAAHannum	0.134	0.070	0.055	0.080	136
EAAGrim	-0.116	0.069	0.090	0.126	136
EAAPheno	0.026	0.063	0.678	0.766	136
DunedinPACE	-0.156	0.080	0.053	0.081	136

[a] Log10 transformed

The p-values were calculated from regression coefficients and standard errors using two-sided z-statistics. False discovery rate (FDR) was used to adjust for multiple comparison. Regression models were adjusted for age, sex, smoking, and methylation beadchip and row. FDR<0.05 are bolded.

Supplementary Table 7. Associations between adipose tissue mtDNA quantity and obesity-related traits.

Outcome	Coef	SE	P-value	FDR	N
Weight	-0.346	0.096	3.2E-04	**0.001**	142
BMI	-0.340	0.095	3.5E-04	**0.001**	142
Waist	-0.320	0.095	0.001	**0.002**	128
WHR	-0.175	0.095	0.066	0.096	66
Fat %	-0.239	0.073	0.001	**0.002**	142
Fat (kg)	-0.357	0.091	9.4E-05	**0.001**	142
Fat-free mass	-0.184	0.061	0.002	**0.006**	142
Ia. Fat[a]	-0.578	0.117	7.5E-07	**2.6E-05**	42
Subcut. Fat[a]	-0.464	0.105	1.1E-05	**1.2E-04**	42
Adipocyte volume	-0.322	0.085	1.6E-04	**0.001**	120
Liver fat %[a]	-0.366	0.123	0.003	**0.006**	84
Total cholesterol	0.076	0.087	0.382	0.432	142
HDL	0.324	0.089	2.7E-04	**0.001**	142
LDL	-0.062	0.092	0.505	0.535	142
Triglycerides[a]	-0.159	0.108	0.142	0.184	142
hsCRP[a]	-0.194	0.109	0.074	0.103	128
Adipsin	-0.229	0.100	0.023	**0.040**	42
Adiponectin	0.357	0.098	2.7E-04	**0.001**	42
ALAT[a]	-0.095	0.099	0.337	0.393	142
ASAT[a]	-0.091	0.092	0.322	0.389	142
BP (systolic)	0.025	0.074	0.739	0.761	138
BP (diastolic)	-0.163	0.085	0.057	0.086	138
Fasting glucose[a]	-0.097	0.085	0.258	0.322	138
Fasting insulin[a]	-0.296	0.103	0.004	**0.009**	134
HOMA-IR[a]	-0.355	0.093	1.4E-04	**0.001**	128
Matsuda[a]	0.345	0.095	2.8E-04	**0.001**	118
LTPA	0.183	0.094	0.051	0.080	130
Sports activity	0.260	0.101	0.010	**0.020**	132
Work activity	-0.068	0.092	0.458	0.501	134
Total activity	0.194	0.093	0.038	0.063	126
EAAHorvath	-0.176	0.073	0.016	**0.030**	136
EAAHannum	-0.273	0.072	1.4E-04	**0.001**	136
EAAGrim	0.145	0.091	0.110	0.148	136
EAAPheno	-0.252	0.054	3.2E-06	**5.5E-05**	136
DunedinPACE	-0.004	0.094	0.964	0.964	136

[a] Log10 transformed

The p-values were calculated from regression coefficients and standard errors using two-sided z-statistics. False discovery rate (FDR) was used to adjust for multiple comparison. Regression models were adjusted for age, sex and smoking. FDR<0.05 are bolded.

Supplementary Table 8. Participant characteristics of the two validation cohorts.

	TwinsUK	Scandinavian T2D-discordant MZ twin cohort
N (twin pairs, singletons)	766* (318, 130)	28 (14, 0)
MZ, DZ full twin pairs	131, 187	14, 0
Age (years), mean (range)	59.3 (38.7–84.6)	68.1 (54-80)
Female, %	100	35.7
Smoking, %	Never 56.7<br>Former 32.4<br>Current 10.1<br>NA: 0.9	Yes 10.7<br>No 53.6<br>NA 35.7
BMI (kg/m^2), mean (range) SD	26.6 (16.2–47.1) 4.9	30.7 (22.3–45.6) 7.0

* Out of which, 535 had DNA methylation data and 765 RNA expression data

DZ = dizygotic; MZ = monozygotic; T2D = type 2 diabetes; NA = not applicable

Supplementary Table 9. Results from the replication analysis.

cg19998400 ~ mtDNA quantity					
Effect	SE	P.value	N, individuals (full MZ,DZ pairs)	Cohort	Smoking-adjusted
-0.120	0.056	**0.032**	28 (14,0)	Scandinavian	No
-0.185	0.029	**3.7E-10**	16 (8,0)		Yes
cg19998400 ~ BMI					
Effect	SE	P.value	N, individuals (full MZ,DZ pairs)	Cohort	Smoking-adjusted
0.003	0.008	0.722	28 (14,0)	Scandinavian	No
0.004	0.012	0.703	16 (8,0)		Yes
0.061	0.008	**1.3E-12**	535 (79,112)	TwinsUK	Yes
cg19998400 ~ *SH3BP4*					
Correlation coefficient		P.value	N, individuals (full MZ,DZ pairs)	Cohort	
0.094		0.662	24 (12,0)	Scandinavian	
0.201		**3.0E-6**	534 (79,111)	TwinsUK	
SH3BP4 ~ BMI					
Effect	SE	P.value	N, individuals (full MZ,DZ pairs)	Cohort	Smoking-adjusted
0.001	0.005	0.907	24 (12,0)	Scandinavian	No
0.010	0.007	0.146	14 (7,0)		Yes
0.072	0.007	**1.1E-22**	765 (131,186)	TwinsUK	No

In addition to smoking status, the linear models were adjusted for age, sex, cohort-specific technical covariates, and family structure and the association assesed with two-tailed hypothesis testing. The correlation between cg19998400 and *SH3BP4* that was determined using the two-tailed Pearson correlation coefficient. P-values<0.05 are bolded.

SH3BP4 = expression of *SH3BP4*

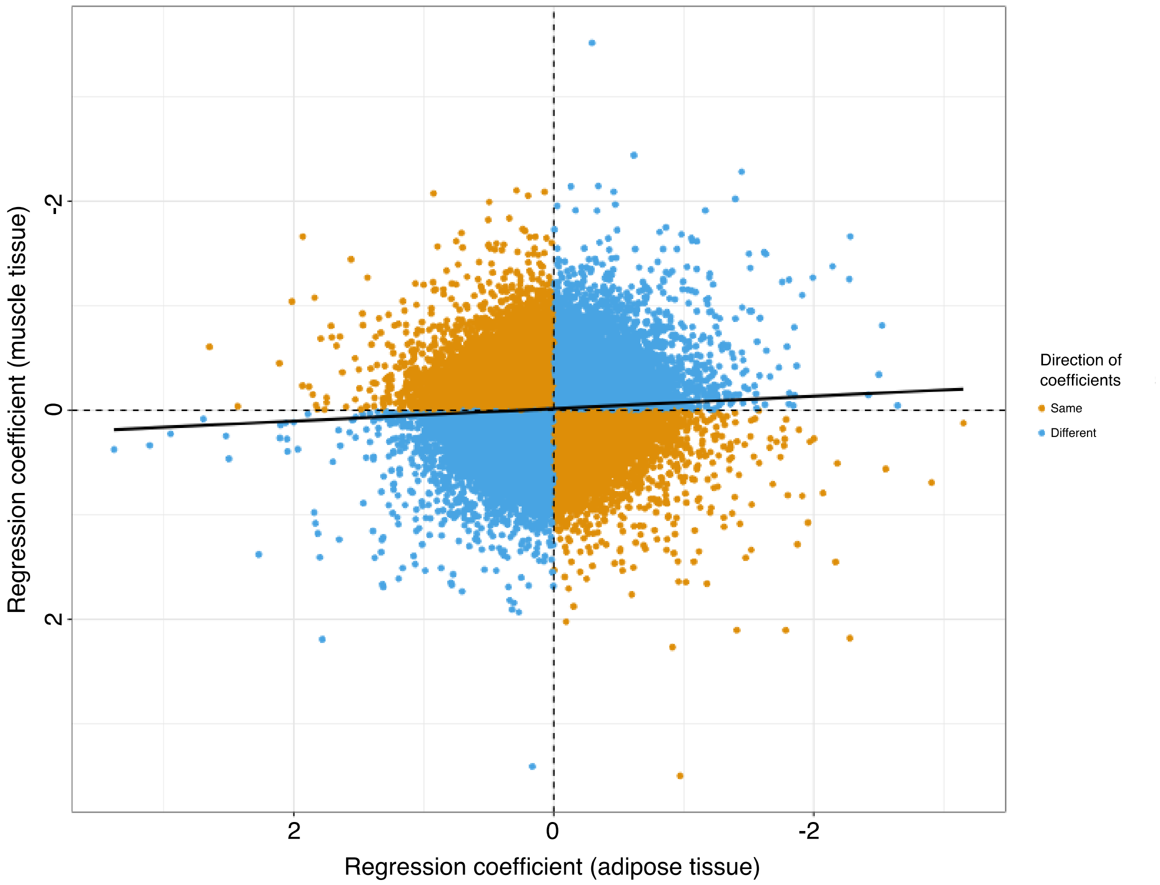

Supplementary Figure 1. **Scatter plot representing the regression coefficients from EWAS on mtDNA quantity in adipose tissue (X) and skeletal muscle (Y).** The black line represents the fitted linear regression line. Yellow points represent CpGs where the regression coefficient had the same direction in both adipose and muscle, whereas blue points indicate CpGs where the direction differed between the two tissues.

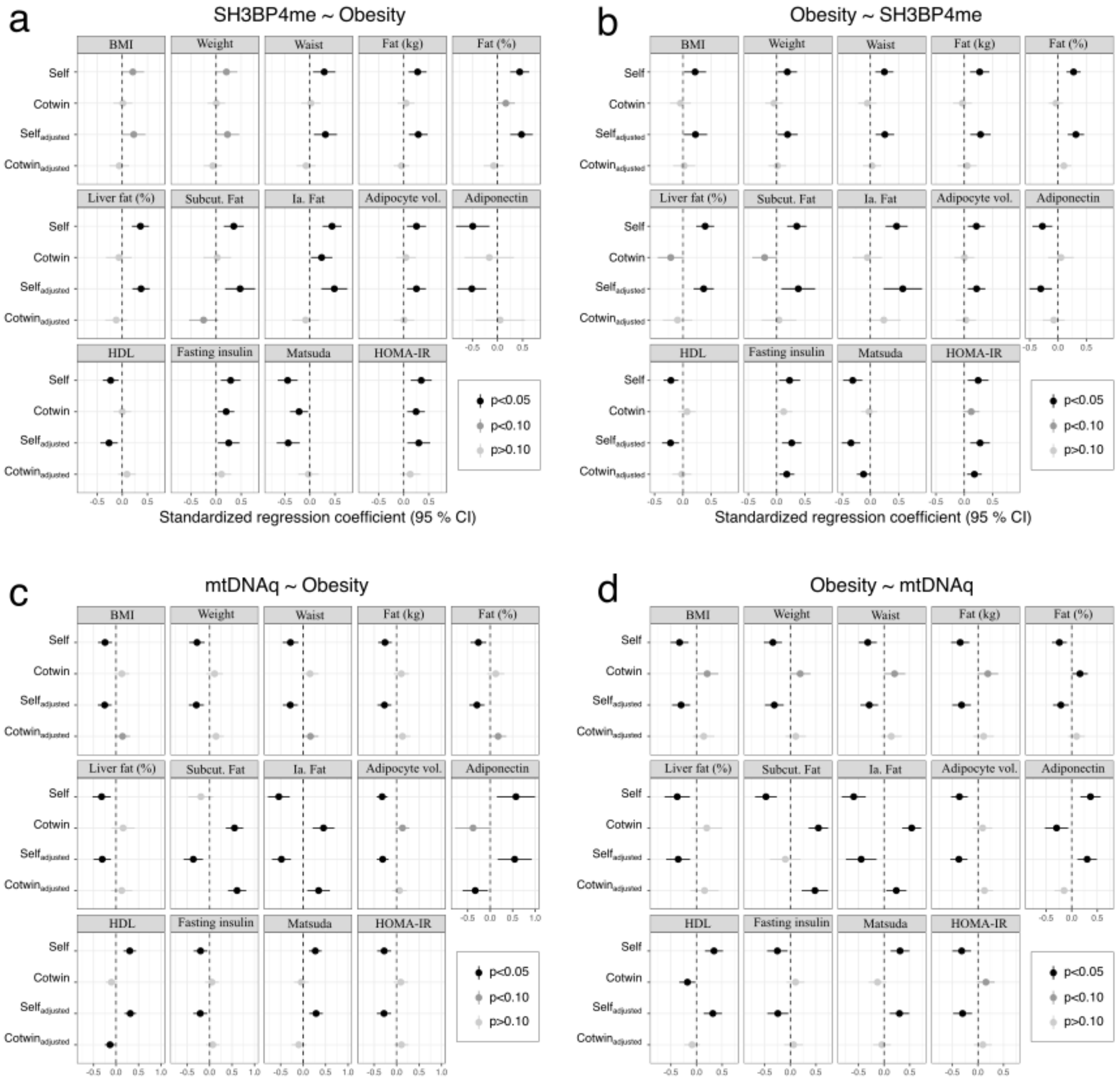

Supplementary Figure 2. **Results from the ICE FALCON analysis, including both monozygotic and dizygotic twin pairs, between adipose tissue mtDNA quantity, *SH3BP4* methylation and obesity-related outcomes. a-d** Point estimates for the standardized regression coefficients and their 95% confidence intervals for the ICE FALCON analysis of **a** obesity-related outcomes regressed against *SH3BP4* methylation, **b** *SH3BP4* methylation regressed against obesity-related outcomes, **c** obesity-related outcomes regressed against mtDNA quantity and **d** mtDNA quantity regressed against obesity-related outcomes. The p-values were calculated from regression coefficients and standard errors using two-sided z-

statistics. No multiple comparison adjustment was applied. Source data with the exact p-values are provided as a Source Data file.

a-b N (BMI, HDL, Weight) = 72 pairs, N (Fat %, Fat kg) = 71 pairs, (Fasting insulin) = 68 pairs, N (HOMA-IR, Waist) = 65 pairs, N (Adipocyte vol., Matsuda) = 60 pairs, N (Liver fat %) = 41 pairs, N (Adiponectin, Ia. Fat, Subcut. Fat) = 21 pairs

c-d N (BMI, HDL, Weight) = 75 pairs, N (Fat %, Fat kg) = 74 pairs, (Fasting insulin) = 71 pairs, N (HOMA-IR, Waist) = 68 pairs, N (Adipocyte vol., Matsuda) = 63 pairs, N (Liver fat %) = 42 pairs, N (Adiponectin, Ia. Fat, Subcut. Fat) = 21 pairs

'Self' represents the association between twin's own X and Y variables whereas 'Cotwin' is the cross-twin cross-trait association i.e. the association between twin's own X variable with their co-twin's Y variable. 'Adjusted' refers to the regression coefficients derived from the ICE FALCON Model 3 that includes both twin's own and their cotwin's X variables.

HDL = high-density lipoprotein; Ia. = intra-abdominal; mtDNAq = mitochondrial DNA quantity, Subcut. = subcutaneous

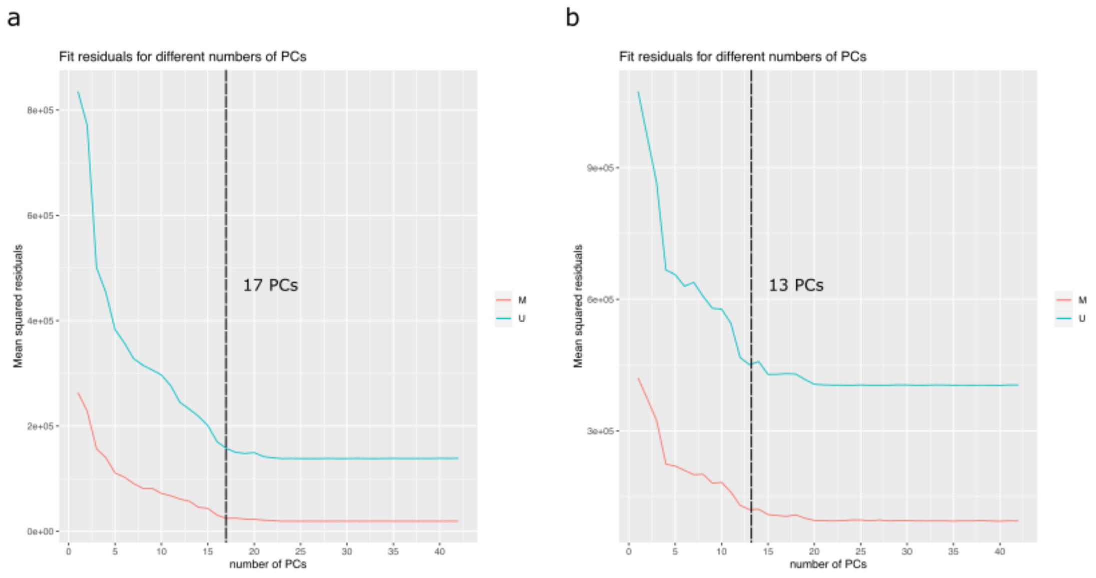

Supplementary Figure 3. **The amount of methylation probe quantile variation unexplained by the control probe principal components under 10-fold cross validation in a adipose tissue and b skeletal muscle.** The number of principal components which the probe quantiles were normalized with is indicated in the plots using a dotted vertical line.

M = methylated probes; U = unmethylated probes

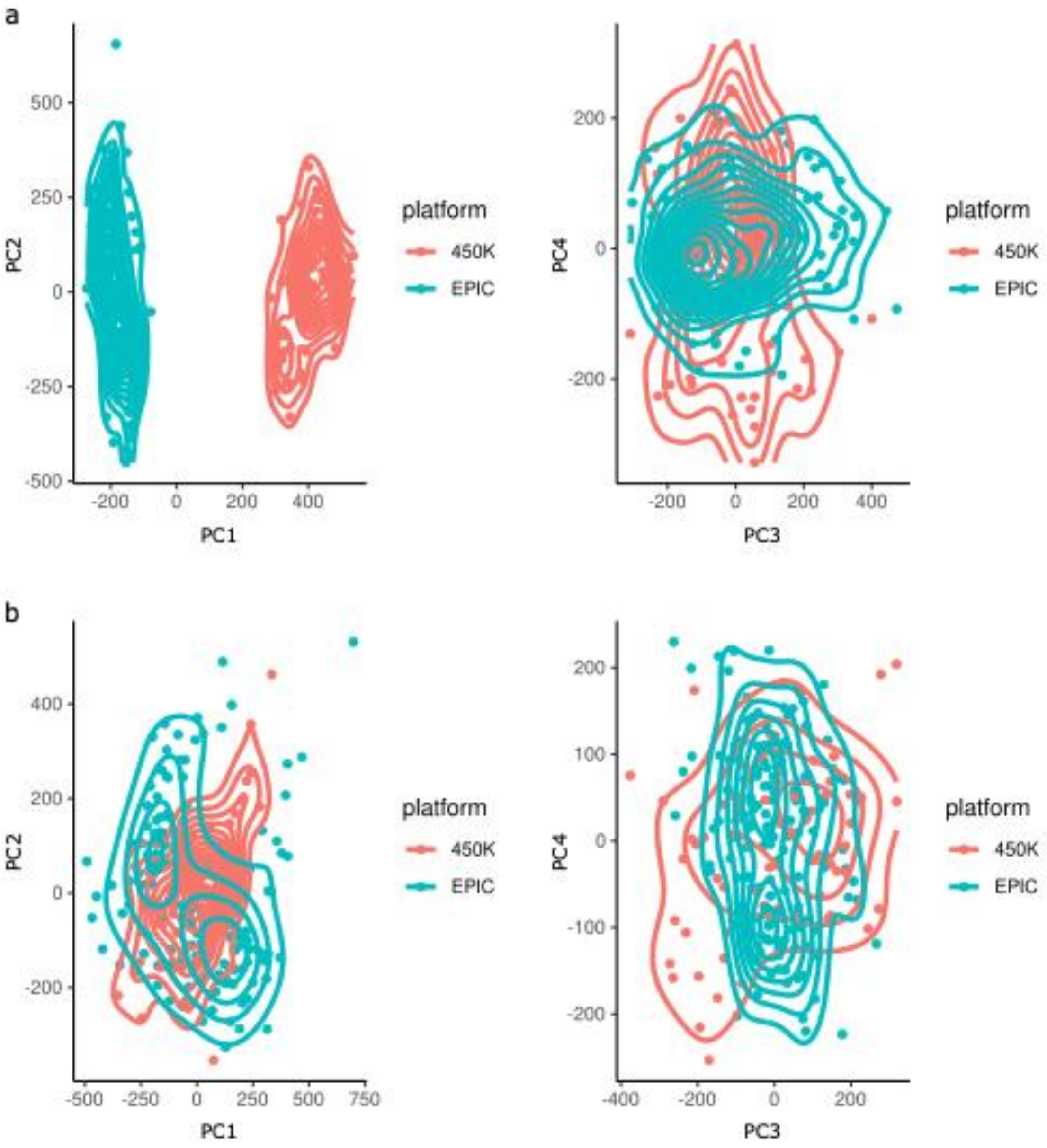

Supplementary Figure 4. **First four principal components of the adipose tissue DNA methylation data indicated by beadchip type (450K/EPIC), a** before and **b** after adjustment with *ComBat*. Each dot represents a methylation sample, and the circles present two-dimensional kernel density estimatates. Red color indicates samples from 450K platform and blue from EPIC platform.
