# Supplementary material for: Twin pair analysis uncovers links between DNA methylation, mitochondrial DNA quantity and obesity
Source: Nat Commun. 2025 May 12;16:4374. doi: 10.1038/s41467-025-59576-7 (PMC12069627; doi:10.1038/s41467-025-59576-7)
Supplement: Supplementary file 2 — Description of Additional Supplementary Files [file 41467_2025_59576_MOESM2_ESM.pdf]

Description of Additional Supplementary Files

File name: Supplementary Data 2
Description: Primer information (oligonucleotide sequences)
