# Supplementary material for: Twin pair analysis uncovers links between DNA methylation, mitochondrial DNA quantity and obesity
Source: Nat Commun. 2025 May 12;16:4374. doi: 10.1038/s41467-025-59576-7 (PMC12069627; doi:10.1038/s41467-025-59576-7)
Supplement: Supplementary file 4 — Reporting Summary [file 41467_2025_59576_MOESM4_ESM.pdf]

nature portfolio

Corresponding author(s): Aino Heikkinen, Miina Ollikainen

Last updated by author(s): Apr 17, 2025

Reporting Summary

Nature Portfolio wishes to improve the reproducibility of the work that we publish. This form provides structure for consistency and transparency in reporting. For further information on Nature Portfolio policies, see our Editorial Policies and the Editorial Policy Checklist.

Statistics

For all statistical analyses, confirm that the following items are present in the figure legend, table legend, main text, or Methods section.

n/a	Confirmed	
☐	☒	The exact sample size (*n*) for each experimental group/condition, given as a discrete number and unit of measurement
☐	☒	A statement on whether measurements were taken from distinct samples or whether the same sample was measured repeatedly
☐	☒	The statistical test(s) used AND whether they are one- or two-sided<br>*Only common tests should be described solely by name; describe more complex techniques in the Methods section.*
☐	☒	A description of all covariates tested
☐	☒	A description of any assumptions or corrections, such as tests of normality and adjustment for multiple comparisons
☐	☒	A full description of the statistical parameters including central tendency (e.g. means) or other basic estimates (e.g. regression coefficient) AND variation (e.g. standard deviation) or associated estimates of uncertainty (e.g. confidence intervals)
☐	☒	For null hypothesis testing, the test statistic (e.g. *F*, *t*, *r*) with confidence intervals, effect sizes, degrees of freedom and *P* value noted<br>*Give P values as exact values whenever suitable.*
☒	☐	For Bayesian analysis, information on the choice of priors and Markov chain Monte Carlo settings
☒	☐	For hierarchical and complex designs, identification of the appropriate level for tests and full reporting of outcomes
☐	☒	Estimates of effect sizes (e.g. Cohen's *d*, Pearson's *r*), indicating how they were calculated

Our web collection on statistics for biologists contains articles on many of the points above.

Software and code

Policy information about availability of computer code

Data collection	Mitochondrial DNA amount: qbase+, Biogazelle, version 3.4.<br>Body composition measures: DEXA, software version 8.8; Lunar Prodigy, Madison, WI, USA; MRI, 1.5 Tesla clinical imager, Avanto/Avantofit, Siemens, Erlangen, Germany; Liver spectra jMRUI 6.0 software<br>Adipocyte diameter: using a custom algorithm for ImageJ (ImageJ 1.42q/ Java 1. 6.0 10 32-bit; https://github.com/birgittavdkolk/vanderkolk_etal_2021)
Data analysis	The data was analyzed using R software (versions 4.2.1 and 4.4.3). Codes used for the data analysis have been deposited to Figshare (https://doi.org/10.6084/m9.figshare.26941927.v3).

For manuscripts utilizing custom algorithms or software that are central to the research but not yet described in published literature, software must be made available to editors and reviewers. We strongly encourage code deposition in a community repository (e.g. GitHub). See the Nature Portfolio guidelines for submitting code & software for further information.

Data

Policy information about availability of data

All manuscripts must include a data availability statement. This statement should provide the following information, where applicable:

- Accession codes, unique identifiers, or web links for publicly available datasets
- A description of any restrictions on data availability
- For clinical datasets or third party data, please ensure that the statement adheres to our policy

The FTC omics data (DNA methylation and RNA sequencing) is part of the 'Twin Study' and deposited with the Biobank of the Finnish Institute for Health and Welfare. For details on accessing the data, see: https://thl.fi/en/research-and-development/thl-biobank/for-researchers/application-process. TwinsUK methylation dataset analysed in the current study is available under ArrayExpress accession number E-MTAB-1866. The expression dataset analysed in the current study is available under EGA accession number EGAS00001000805. All additional data access requests are overseen by the TwinsUK Resource Executive Committee (TREC). For information on access to these data and how to apply, see https://twinsuk.ac.uk/researchers/access-data-and-samples/request-access/
The Scandinavian T2D-discordant cohort of MZ twins data (accession number LUDC2020.08.14) are deposited in the Lund University Diabetes Centre repository (https://www.ludc.lu.se/resources/repository) and while summary data and look-ups are available to academic researchers upon request through the repository portal, individual level data are not available due to ethical and legal restrictions related to Swedish Biobanks in Medical Care Act and European GDPR legislation. The EWAS summary statistics on mtDNA quantity in adipose and muscle tissues, produced in this study is deposited to Figshare (https://doi.org/10.6084/m9.figshare.26941927.v3). Source data are provided as Source data file within the manuscript.

Research involving human participants, their data, or biological material

Policy information about studies with human participants or human data. See also policy information about sex, gender (identity/presentation), and sexual orientation and race, ethnicity and racism.

Reporting on sex and gender	Self-reported sex is considered as a covariate in the study. No sex-specific analyses were conducted, and the results apply to both sexes. No disaggregated data on sex can be provided with the manuscript due to restrictions on sharing individual-level data.
Reporting on race, ethnicity, or other socially relevant groupings	We did not perform any race or ethnicity specific analyses in the manuscript. The main cohort involved participants recruited from Finnish population-based cohorts, and the validation cohort include community-dwelling adult twins in the United Kingdom (TwinsUK), and Sweden or Denmark (Scandinavian T2D-discordant MZ cohort).
Population characteristics	Table 1 in the manuscript presents the detailed population characteristics. The mean age was 45.7 years (range 22.8 - 69.3) and 59 % were female. The study encompasses 163 and 10 monozygotic and dizygotic twins, respectively.
Recruitment	The participants were recruited from a population-based Finnish Twin Cohort, targeting MZ twin pairs to study obesity-related metabolism based on their self-reported BMI to ensure the presence of twin pairs with varying discordance for BMI, as described in the manuscript.
Ethics oversight	The Ethics Committee of the Hospital District of Helsinki and Uusimaa approved the protocols of the data collections, and all participants provided their written informed consent.

Note that full information on the approval of the study protocol must also be provided in the manuscript.

Field-specific reporting

Please select the one below that is the best fit for your research. If you are not sure, read the appropriate sections before making your selection.

☒ Life sciences ☐ Behavioural & social sciences ☐ Ecological, evolutionary & environmental sciences

For a reference copy of the document with all sections, see nature.com/documents/nr-reporting-summary-flat.pdf

Life sciences study design

All studies must disclose on these points even when the disclosure is negative.

Sample size	Total sample size in the study was 173 individuals. The sample size varied between the tissues and analyses, and therefore sample-specific sample sizes are reported in the manuscript. The sample size was not predetermined but we used all available samples to have the largest sample size possible.
Data exclusions	There were no specific inclusion or exclusion criteria in the study. All recruited study participants who consented to adipose and/or muscle tissue biopsies are included. All DNA methylation and mitochondrial DNA quantity data samples were included if the samples passed the QC as described in the manuscript.
Replication	The replication of the study findings on the association between mtDNA quantity, BMI and methylation and expression of SH3BP4 were succesflly replicated in TwinsUK and Scandinavian T2D-discordant MZ twin cohorts.
Randomization	The allocation of samples into groups was not applicable in this study.

Blinding	The study did not involve any blinding protocols.

Reporting for specific materials, systems and methods

We require information from authors about some types of materials, experimental systems and methods used in many studies. Here, indicate whether each material, system or method listed is relevant to your study. If you are not sure if a list item applies to your research, read the appropriate section before selecting a response.

Materials & experimental systems

n/a	Involved in the study
☒	☐ Antibodies
☒	☐ Eukaryotic cell lines
☒	☐ Palaeontology and archaeology
☒	☐ Animals and other organisms
☒	☐ Clinical data
☒	☐ Dual use research of concern
☒	☐ Plants

Methods

n/a	Involved in the study
☒	☐ ChIP-seq
☒	☐ Flow cytometry
☒	☐ MRI-based neuroimaging

Plants

Seed stocks	*Report on the source of all seed stocks or other plant material used. If applicable, state the seed stock centre and catalogue number. If plant specimens were collected from the field, describe the collection location, date and sampling procedures.*
Novel plant genotypes	*Describe the methods by which all novel plant genotypes were produced. This includes those generated by transgenic approaches, gene editing, chemical/radiation-based mutagenesis and hybridization. For transgenic lines, describe the transformation method, the number of independent lines analyzed and the generation upon which experiments were performed. For gene-edited lines, describe the editor used, the endogenous sequence targeted for editing, the targeting guide RNA sequence (if applicable) and how the editor was applied.*
Authentication	*Describe any authentication procedures for each seed stock used or novel genotype generated. Describe any experiments used to assess the effect of a mutation and, where applicable, how potential secondary effects (e.g. second site T-DNA insertions, mosiacism, off-target gene editing) were examined.*
